# The role of palliative radiotherapy for haemostasis in unresectable gastric cancer: a single-institution experience

**DOI:** 10.3332/ecancer.2014.384

**Published:** 2014-01-10

**Authors:** Cheng Lee Chaw, Paddy G Niblock, Cheng Shu Chaw, Douglas J Adamson

**Affiliations:** 1 Princess Alexandra Centre, Oncology Department, Ninewells Hospital & Medical School, Dundee DD1 9SY, Scotland, UK; 2 Faculty of Applied Science, Department of Pharmacy, Health and Well Being, University of Sunderland SR1 3SD, UK

**Keywords:** bleeding, gastric cancer, haemostasis, palliative, radiotherapy

## Abstract

**Purpose:**

To evaluate the outcomes of patients with gastric cancer bleeding who had been treated with palliative radiotherapy with haemostatic intent.

**Methods and materials:**

Fifty-two gastric cancer patients aged 52–92 years (median 78 years) with active bleeding or anaemia resulting from inoperable gastric cancer were treated with short-course radiotherapy. Responses to radiotherapy treatment were evaluated based on the changes of haemoglobin level, number of transfusions received before and after radiotherapy, and overall median survival.

**Results:**

Thirty-nine (75%) patients received single 8 Gy fraction, and 13 (25%) patients received 20 Gy in five daily fractions. The need for transfusion was evaluable in 44 patients, and the response rate was 50%, with less requirement for blood transfusions within four weeks of radiotherapy. There was also an increase in mean haemoglobin level (0.66 ± 1.12 g/dl, *p *< 0.01) after radiotherapy in 35 evaluable patients. The overall median survival (calculated from last day of treatment to date of death) was 160 days (95% CI of 119–201 days), making actuarial 12-month survival 15%.

**Conclusion:**

Palliative short-course radiotherapy is a reasonably effective treatment that can provide durable palliation of bleeding in gastric cancer.

## Introduction

Gastric cancer is the fourth most common malignancy in men and the fifth most common malignancy in women globally. It accounts for 8% of the total number of cases of cancer and 10% of annual deaths from cancer worldwide [1]. The current curative treatment for gastric cancer is surgery, but most patients are diagnosed with locally advanced or metastatic disease at presentation. The prognosis of these patients is poor, and a five-year survival rate of 10–20% has been reported [2, 3].

The local symptoms caused by gastric cancer are pain, obstruction (dysphagia, vomiting), and bleeding (melaena, haematemesis). Overt gastrointestinal bleeding from gastric cancer at presentation is uncommon but does occur in up to 10% of patients [4], and this can be a very distressing symptom for both patients and their family members. Chronic bleeding from gastric cancer can cause anaemia and subsequently fatigue and anorexia. Anaemia can also interfere with symptom control, and control of bleeding is important to improve the quality of life in these patients [5].

There are several treatment modalities for gastric bleeding, including palliative surgical resection, endoscopic treatment, and radiotherapy, but little research into which treatment is the most effective option has been performed.

Gastric cancer bleeding can be relieved by palliative resection; however, only fit patients can undergo surgery, and it is associated with significant mortality approaching 10% as well as morbidity in about a third of patients [6–8]. Surgery may provide rapid relief from symptoms, but prolongation of survival is noted in only 6% of patients and improvement in the quality of life following palliative resection is unclear [7].

Endoscopic intervention including argon plasma coagulation has been reported to achieve haemostasis in 67% of patients with gastroduodenal tumour bleeding [9]; however, this is associated with risk of perforation (5–15%), and the re-bleeding rate is high [9].

Radiotherapy has been used to palliate bleeding from other malignant tumours such as lung, cervical, and bladder cancers [10, 11]. Radiation causes denudation of the intima of the blood vessels supplying the tumour, leading to capillary necrosis and the formation of thrombosis, which blocks the lumen of blood vessels, achieving haemostasis [4]. The response rate to this treatment has been reported to be as high as 50–90% [2–4, 12, 13], but the retrospective nature of these reports needs to be taken into account.

Here, we report the results of a single-centre retrospective study on ‘short-course’ radiotherapy to palliate patients with symptomatic locally advanced gastric cancer and evaluate its effectiveness in relieving symptoms of bleeding or anaemia.

## Methods and materials

### Patients

Two hundred and ninety-eight consecutive patients who were diagnosed with gastric cancer between May 2001 and December 2010 and referred for oncological treatment were analysed retrospectively. The patients regarded as having gastric cancer on endoscopy findings and who had a pathological diagnosis of only high-grade dysplasia were included. Furthermore, those patients who presented with gastrointestinal bleeding or anaemia and were treated with short-course palliative radiotherapy were eligible for inclusion in this retrospective study. The diagnosis of gastrointestinal bleeding was established either by endoscopic findings or from the clinical symptoms of patients (melaena or haemetemesis) supported by evidence of a low or falling haemoglobin level. In our region, palliative radiotherapy to the stomach alone is generally reserved for patients unfit for other treatment strategies such as chemotherapy or surgery.

The patients’ gender, age, histology, stage, and treatment factors (use of chemotherapy before and after radiation treatment, postradiation transfusion and mean haemoglobin level before and after radiotherapy) as well as radiotherapy parameters (field arrangements, total dose, dose fractionation regimens) were extracted from the patient records.

### Treatment procedures

All patients with gastrointestinal bleeding received external beam radiotherapy using high-energy (6 or 10 MV) photons from a linear accelerator. The patients were treated supine with parallel-opposed pair of anterior/posterior fields, and the radiotherapy was delivered either as a single fraction of 8 or 20 Gy in five daily fractions over one week. The 20 Gy treatment in five fractions was usually given to patients with a better performance status or those who had dysphagia caused by tumour obstructing the lower oesophagus.

### Response assessment

The efficacy of the short-course radiotherapy was evaluated. With reference to previous studies by other groups [2, 4, 12, 13], we defined a positive radiotherapy response if

- patients subsequently required a blood transfusion but did not need one in the four weeks after radiotherapy was given (‘transfusion group’) or
- patients did not require transfusion at all and survived for three months or more (‘Non-transfusion group’).

We also evaluated the efficacy of the short-course radiotherapy on the basis of improvement in haemoglobin levels in patients after the radiotherapy.

The difference between the mean haemoglobin values before and after radiotherapy was therefore reviewed. The mean preradiotherapy haemoglobin level was obtained from the blood test results from the day of admission to hospital with gastrointestinal bleeding to the first fraction of radiotherapy. Hence the value reflects the underlying disease and effects of transfusion only. The mean postradiotherapy haemoglobin level reflects the four-week period starting with the last fraction of radiotherapy. This parameter was used to measure the effect of radiotherapy: a decrease from the mean preradiotherapy haemoglobin level was considered to be indicative of re-bleeding.

We were also keen to determine the overall survival of this group of patients, which we calculated from the day the patient received the last fraction of radiotherapy.

### Statistical analysis

Statistical analyses were carried out with the SPSS (Statistical Package for Social Sciences) version 18. Survival was estimated by the Kaplan–Meier method. Univariate analysis with log rank test was performed to study different factors correlating with survival. The alteration of haemoglobin levels was analysed with paired *t*-test; *p*-values <0.05 were considered to be statistically significant.

## Results

Sixty-four of the 298 patients were referred for palliative radiotherapy for haemostatic intent. However, 12 of the 64 patients did not receive palliative radiotherapy after the initial consultation either because the oncologist assessed that they were not fit for treatment or there was no immediate indication for the treatment. Fifty-two patients received palliative radiotherapy for control of bleeding symptoms of locally advanced gastric cancer. The clinical details of these 52 patients are summarised in Table 1. The median age of the patients was 78 years (range 52–92 years); they were predominantly male (*n *= 34, 65%). The median age for the male patients was 76 years (range 52–90 years), and the median age for the female patients (*n* = 18, 35%) was 81 years (range 61–92 years).

Thirty-nine (75%) patients received a single 8 Gy treatment and 13 (25%) patients received 20 Gy in five daily fractions. Two patients were subsequently re-treated with palliative radiotherapy (both re-treatments were with an 8 Gy single fraction). One of them received 8 Gy single fraction on two occasions for haemostatic intent, and these radiotherapy treatments were given eight months apart. The other patient was initially treated with 20 Gy in five fractions for palliation of dysphagia, and further radiotherapy treatment was given one year later for haemostatic intent. There were seven (14%) patients treated with systemic chemotherapy before receiving short-course radiotherapy for controlling the bleeding symptom, and only three (6%) patients received systemic treatment after palliative radiotherapy for control of the disease, as would be expected for an unfit cohort of patients.

### Treatment outcomes

**Response to radiotherapy**

Eight of the 52 patients had received palliative radiotherapy for haemostasis but did not have any documentation of transfusion data. Hence the response to short-course treatment could not be evaluated in these patients. The 44 remaining evaluable patients were categorised into two groups in terms of transfusion requirement (Table 2).

**Non-transfusion group: **Fifteen patients did not receive any transfusion after palliative radiotherapy. One patient died within a week and six patients died within a month. The remained eight patients survived beyond three months and did not require transfusion; hence the response rate to radiotherapy in this group was 53% (8/15).

**Transfusion group: **Twenty-nine patients required postradiation transfusion. Of the 29 patients, fifteen (52%) required transfusion within four weeks; hence palliative radiotherapy was deemed ineffective. Fourteen of the 29 patients required transfusion at least a month or more after radiotherapy. Hence, the ‘response rate’ to radiotherapy was 48% (14 of 29). We also reviewed the number of transfusions that patients required until they died (Table 2). Thirteen (45%) of the 29 patients required one transfusion before they died, and the rest required multiple transfusions before death.

Thus, 22 of 44 patients (50%), that is, 14 from transfusion group and 8 from non-transfusion group, responded to radiotherapy.

**Mean haemoglobin difference before and after radiotherapy**

**Figure 1.**
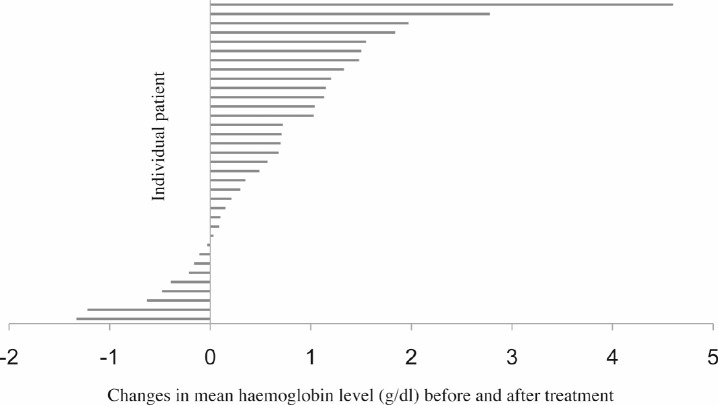
Differences in mean haemoglobin level before and after palliative radiotherapy in 35 patients. Twenty-six patients had an increase in mean haemoglobin level after treatment, whereas nine patients had a fall. The overall increment in mean haemoglobin level was 0.66 ±1.12g/dl in 35 patients, *p* < 0.01.

Only 35 patients had complete preradiotherapy and postradiotherapy haemoglobin levels recorded, and in these patients, the differences in mean values were evaluated. The blood results of the remaining 17 patients were not evaluable due to incomplete documentation. The mean haemoglobin value before palliative radiotherapy for these 35 patients was 9.53 g/dl, whereas the mean haemoglobin level after radiotherapy treatment was 10.2 g/dl. There was a statistically significant improvement in mean haemoglobin level after radiotherapy compared with the preradiotherapy haemoglobin (*p* < 0.01, Figure 1).

### Median survival

**Figure 2.**
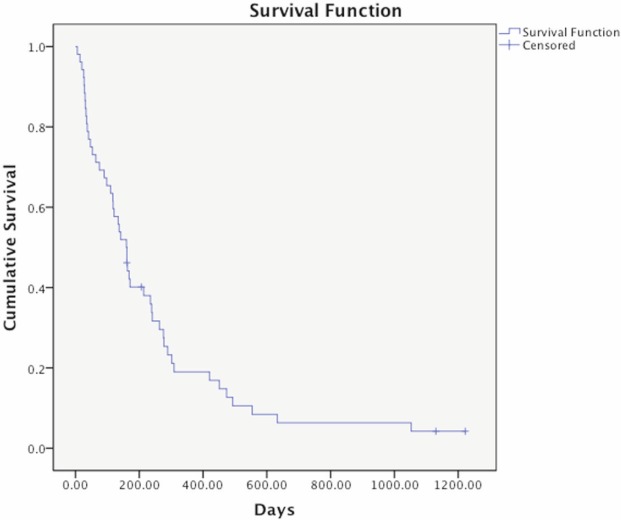
Kaplan–Meier survival curve of 52 patients receiving palliative radiotherapy. The overall median survival of these patients was 160 days (95% CI 119–201 days). Four patients were alive and censored at the time of analysis.

The overall median survival of patients in this study was 160 days (95% CI of 119–201 days) ranging from 6 days to 1053 days. At the time of analysis, four patients were still alive (Figure 2). The median survival of those 12 patients who did not receive palliative radiotherapy was 49 days (95% CI of 18–80 days).

The survival rates at 3, 6, and 12 months were 65%, 23%, and 15%, respectively. Univariate analysis on different pretreatment factors (stage, histology, radiotherapy dose, etc.) that might have an impact on survival was performed, but as shown in Table 3, no statistically significant difference was found.

## Discussion

There are five retrospective studies in recent years that have described the benefits of using radiotherapy to palliate bleeding in patients with unresectable gastric cancer [2–4, 12, 13]. The response rates for controlling bleeding in these studies varied from 50–90%, such wide range is due to the difference in the definition of treatment success. The reported overall median survival with palliative radiotherapy varied from 3–5.2 months (Table 4). These retrospective studies, however, had relatively small sample sizes of 19–37 patients, and no randomised controlled trial to evaluate the efficacy of short-course radiotherapy on the palliation of symptomatic gastric cancer has been published.

Our study evaluated the outcomes of 52 patients with gastric cancer bleeding treated with radiotherapy in a single regional centre over a ten-year period, with the sample size larger than those reported previously [2–4, 12, 13]. Like all retrospective works, the results need to be interpreted with caution. It is difficult to obtain retrospective data for each patient in a standard way, and the information on performance status or symptom improvement may be hard to obtain in all cases. The strengths of our series are that all patients in our catchment population (approximately 400,000) were treated in a single oncology radiotherapy centre by only a few consultants using a similar technique. We therefore believe that these results reflect outcomes typical for all-comers within a population rather than subgroups selected for study, for example, in clinical trials with specific exclusion criteria. We believe case ascertainment to be high for similar reasons of easy data retrieval from the comprehensive and mainly electronic records of one regional Scottish centre.

The majority (73%) of the patients were elderly patients over 70 years of age and were unfit to receive radical treatment or palliative systemic treatment. A small number of patients in our study had been diagnosed with early-stage disease (stages I and II) but owing to significant co-morbidities were fit neither for surgery nor for palliative chemotherapy; therefore palliative radiotherapy was given for patients who were symptomatic with bleeding. As none of our patients received concurrent chemoradiation treatment, and only 14% of patients (7 of 52) received chemotherapy prior to radiotherapy, this has provided a useful description of the natural history of patients who are treated with radiotherapy alone. Six percent (3 of 52) of these patients received systemic treatment after radiotherapy. The overall median survival in our group of 160 days was unlikely to be confounded by the use of other treatments, and this result was similar to the result shown by Tey *et al *[12].

Different dose fractionation schedules varying from single 8 Gy to dose of 50 Gy in 25 fractions have been published in previous papers [2–4, 12, 13]. Two of the studies suggested the use of higher biological effective dose (BED) of over 39 Gy [2, 3] would provide better local control and also survival. Tey *et al *suggested that there was no statistically significant difference in median survival between lower and higher BED, but the result favoured the use of higher dose, which tended to confer longer survival [12]. Our study could lend support to this, as the median survival of the 39 patients receiving a single 8 Gy versus the 13 patients treated with 20 Gy in five fractions was 142 versus 239 days, respectively, *p *= 0.202 (Table 3); however, this finding is likely be confounded by the selection of patients with better performance status for the longer fractionation and by the preferences of the radiotherapist. Assuming alpha/beta (*α*/*β*) ratio was 10, the BED in our study ranged from 14.4 Gy (8 Gy single fraction) to 28 Gy (20 Gy in five fractions), which was at least 1.5 times less than the dose applied in the previous studies [2, 3, 12, 13], although the results appear similar. Our study has shown that the use of lower dose treatment has shown a similar median survival compared with higher dose treatment in this, generally unfit, patient cohort.

Previous studies evaluated the response of radiotherapy by reviewing the pre- and postradiotherapy haemoglobin levels and also the reduction of transfusion units or amount of transfused blood after radiotherapy [2, 4, 13]. However, it is unclear from these studies at what stage and how often after radiotherapy, the haemoglobin level was checked. Lee *et al *had commented on the challenging aspects of assessing the efficacy of radiotherapy [4]. This correctly states that a single haemoglobin level as an end point to evaluate response is flawed, as haemoglobin level does not reflect acute gastrointestinal bleeding directly, and because the level of haemoglobin varies with gender and also comorbidity. Therefore, Lee *et al*. had suggested the use of mean value of haemoglobin level and mean value of reduction in transfusion units required postradiotherapy for a period of three months. A mean haemoglobin level is more likely to reflect the true effect of radiotherapy.

We evaluated the response to treatment by calculating the mean value of haemoglobin levels preradiotherapy and postradiotherapy. There was a statistically significant increase in the level of haemoglobin after radiotherapy. This finding was comparable with the results reported in the previous studies (Table 4), which showed an increase in the level of postradiotherapy haemoglobin although the values did not return to the reference range in all cases. The likely explanation was that anaemia persisted because of underlying disease and comorbidity of patients, even though radiotherapy has achieved haemostatic effects.

Most studies deemed radiotherapy treatment effective if patients did not require transfusion within four weeks or more after radiotherapy [2, 4, 13] in which the cutoff point of four weeks seems arbitrary. For the purpose of comparison of response to short-course radiotherapy, we adopted the similar approach. We assessed the response of short-course radiotherapy by recording the need of transfusion within four weeks after completing radiotherapy treatment. In our study, 15 patients did not require transfusion; however, half of this non-transfusion group died within a month; therefore the radiotherapy treatment was not considered effective. For the ‘transfusion group’ (*n *= 29), which comprised patients receiving transfusion within four weeks after radiotherapy, nearly half of them (13 of 29) required only one transfusion before they died. The rest required multiple transfusions and three patients of this group required transfusion on more than five occasions, so it is evident that the survival of these patients was prolonged by the transfusion treatment, but initial haemostasis was achieved by radiotherapy. It is notable that in a group perceived to have a dire outlook, almost one in seven patients were alive one year after completing radiotherapy, and it may be that this simple treatment provides excellent palliation at small cost to the individual.

To date, there are no accurate measurable parameters to evaluate the effectiveness of radiotherapy for gastric cancer bleeding. Theoretically, haemostatic effects can be accurately assessed by direct view of the bleeding source through an endoscopic approach in conjunction with examination of the haemoglobin level at regular intervals. However, this approach is not suitable or particularly helpful in patients who have advanced disease. In our retrospective study, the change in mean haemoglobin and also the requirement of transfusion were used as surrogates to assess efficacy of treatment.

In conclusion, our study suggests that radiotherapy is a good option for patients with gastric cancer bleeding as shown by the overall median survival and the improvement of mean haemoglobin levels after radiotherapy. Either a single 8 Gy fraction or 20 Gy in five fractions, corresponding to a BED of 14.4to 28 Gy, appears to provide similar response rates to those treatments of higher BED (Table 4). Further investigations with control group not receiving radiotherapy for palliation of gastric cancer bleeding and larger sample size would provide more evidence of the efficacy of this treatment in controlling symptomatic bleeding, and as in most palliative treatments, it would be important to assess the effect of treatment on quality of life, although such data are difficult to collect from patients who are frail and unfit.

RT: radiotherapy; CRT: chemoradiation; Hb: haemoglobin; 2D: two dimensional; 3D: three dimensional; CT: computed tomography; BED: biological effective dose; PA: posterior-anterior; L: lateral

## Conflict of interest notification and funding disclosure

All the authors of this manuscript declare that there are no financial disclosures or conflicts of interest that could be perceived as prejudicing the impartiality of the research reported.

## Figures and Tables

**Table 1.** Patients’ demographics.

		Number (n)	Percentage (%)
Gender	Male	34	65
	Female	18	35
Age (years old)	51–60	5	10
	61–70	8	15
	71–80	19	37
	81–90	19	37
	> 91	1	2
Disease Status	Stage I	2	4
	Stage II	4	8
	Stage III	13	25
	Stage IV	23	44
	Biopsy proven, staging not documented	7	14
	Presumed gastric carcinoma, staging not documented	3	6
Histology	Poorly differentiated adenocarcinoma	27	52
	Mucinous adenocarcinoma	1	2
	Signet ring adenocarcinoma	6	12
	Carcinoma with neuroendocrine features	3	6
	Intestinal type adenocarcinoma	4	8
	High-grade dysplasia	2	4
	Moderately differentiated adenocarcinoma	7	14
	Anaplastic carcinoma	1	2
	Squamous cell carcinoma	1	2
Pre-radiotherapy chemotherapy	Yes No	7 45	14 86

**Table 2.** Number of transfusions required by 44 patients before they died.

Category	Number of transfusions required	Number of patients
Non Transfusion group	0	15
Transfusion group	1 2 3 4 7 8 10	13 4 5 4 1 1 1
**Total no. of patients**		44

8 patients did not have documentation of transfusion, hence were not included in this analysis

**Table 3.** Factors that could potentially have an impact on median survival time.

Factors	Median survival (days)	*p* value
**Gender**		
· Female	137	0.97
· Male	160	
**Disease status**		
· Stage I	163	
· Stage II	420	
· Stage III	121	0.73
· Stage IV	137	
· Biopsy proven gastric carcinoma, no staging documented	160	
· Presumed gastric carcinoma, no staging documented	241	
**Histology**		
· Poorly differentiated adenocarcinoma	161	
· Mucinous adenocarcinoma	276	
· Signet ring adenocarcinoma	168	
· Carcinoma with neuroendocrine features	277	0.068
· Intestinal type adenocarcinoma	47	
· High grade dysplasia	241	
· Moderately differentiated adenocarcinoma	110	
· Anaplastic carcinoma	171	
· Squamous cell carcinoma	474	
**Radiotherapy regimen**		
· 8 Gy	142	0.202
· 20 Gy in 5 fractions	239	
**Chemotherapy given prior to radiation treatment**		
· Yes	110	0.128
· No	161	
**Chemotherapy given after radiation treatment**		
· Yes	289	0.487
· No	142	
**Age Group**		
· 51–60	110	
· 61–70	90	0.209
· 71–80	161	
· 81–90	160	

**Table 4.** Summary of the 6 retrospective studies on efficacy of palliative radiotherapy treatment in advanced gastric cancer.

Studies	Number of patients received palliative RT(n)	Patients treated with CRT or pretreated with chemotherapy	RT technique and dose/fractionations and biological effective dose	Treatment outcomes				
				Medianoverallsurvival(months)	Responserate (%)	Mean Hb levelpre and postRT (g/dl)	Transfusion unit/volume before and after RT treatment	Effectiveness of BED (Gy α/β) in palliation of gastric cancer
Tey J, *et al* 2007	33	No patient received CRT	CT 3D planning, 6-10 MV photons, 8 Gy single fraaction, 20 Gy in 5 fractions, 30 Gy in 10 fractions, 30 Gy in 12 fractions, 35 Gy in 14 fractions, 37.5 Gy in 15 fractions, 40 Gy in 16 fractions.	4.8	Response to bleeding: 54^*^	Not reported	Not reported	No statistical significant difference in local control of symptom between patients received BED < 39 Gy_10_ and those received BED >39 Gy_10_
Kim M, *et al* 2008	37	24 patients received CRT.	Planning techniques not reported, 35 Gy in 14 fractions	5.2 (Median overall survival were higher in CRT arm than RT alone (6.7 months vs. 2.4 months, *p* = 0.08)	Responseto bleeding: 70^*^	Not reported	Not reported	BED 41 Gy_10_ conferred better local control of symptoms but not overall survival
Hashimoto K, *et al* 2009	19 patients in the study but only 13 completed RT treatment	4 patients received CRT, 16 patients received chemotherapy prior to palliative RT	CT 3D planning, 6-25 MV photons, 20 Gy in 10 fractions to 50 Gy in 25 fractions.	3.4	68^**^	Hb level before RT ranged from 3.5 - 8.4 compared to rangeof 6.4-12.3 (Mean Hb level was not reported)	Range of 700-4600 ml were used prior RT No data of transfusion reported after RT.	BED 50 Gy_10_ conferred higher success rate ofhaemostasis compared to those received BED <50 Gy_10_
Lee JA, *et al *2009	23	No patient received CRT, 14 received palliative chemotherapy prior to palliative RT	2D planning, barium contrast to aid visualisation of organ motion, 10-15 MV photons, 30-44 Gy in 10-22 fractions	Not reported	91% achieved complete resolution of symptoms ^***^	9.1 compared to 10.6, *p* < 0.001	9.5 ± 6.51 units compared to 2.8±6.8, *p* < 0.001	Not reported
Asakura H, *et al *2011	30	12 patients received CRT, 21 patients received chemotherapy prior to palliative RT	CT 3D planning, 2-18 MV photons, 21 Gy in 7 fractions, 27 Gy in 9 fractions, 30 Gy in 10 fractions	3.6	73^****^	4.9 compared to 8.2 (1 month before and after the last blood transfusion) *p* < 0.0001 (Mean Hb level was not reported)	2236 ml compared to 273 ml, *p* < 0.0001	BED 39 Gy_10_, conferred a response rate comparable to higher BED(50 Gy_10_ or more)

Abbreviations: RT, radiotherapy; CRT, chemoradiotherapy; Hb, haemoglobin; 2D, two dimensional; 3D, 3 dimensional; CT, computed tomography; BED, biological effective dose; α/β ratio: 10Gy; AP, anterior-posterior; PA, posterior-anterior; L, lateral

* Both manuscripts reported response to RT based on the evaluation of patient symptoms on follow up period with no requirement of further treatments.

** The manuscript described treatment success as patient being alive with no requirement for blood transfusion after 1 month or more following RT.

*** The manuscript described positive response to RT as complete resolution of symptoms based on subjective evaluation.

**** Author described treatment success as patient did not require blood transfusion for 1 month or more after beginning of radiotherapy.
